# Einfluss der Verschmutzung von Reflexionsfolien auf ihr Reflexionsverhalten

**DOI:** 10.1007/s10341-022-00799-z

**Published:** 2023-01-04

**Authors:** Stephan Bell, Achim Kunz, Lutz Damerow, Michael Blanke

**Affiliations:** INRES-Gartenbauwissenschaften, Universität Bonn, Bonn, Deutschland

**Keywords:** Apfel (*Malus domestica* Borkh.), Anthozyansynthese, Color-up, Extenday, Fruchtfarbe, Lichtreflexion, Mulchfolie, Mylar, Nachhaltigkeit, Oberflächenrauheit, Apple (*Malus domestica* Borkh.), Anthocyanin synthesis, Fruit colour, Light reflection, Mulch, Mylar, Resource conservation, Sustainability, Surface roughness

## Abstract

Ziel der Untersuchungen war es, den Einfluss einer Verschmutzung von Reflexionsfolien, die zur Verbesserung der Fruchtfarbe eingesetzt werden, hinsichtlich ihres Reflexionsverhaltens zu untersuchen, was sich auf ihre Nutzungsdauer bzw. Wiederverwendbarkeit auswirkt. Dazu wurden zwei Reflexionsfolien, eine silberne Alufolie und eine weiße Polypropylen Gewebefolie (Lumilys™, Beaulieu, Belgien) – wie nach einem Herbststurm – unterschiedlich stark verschmutzt; saubere Folien dienten als Kontrolle.

Mit einem Spektrometer der Firma StellarNet (Tampa, FL, USA) wurden die senkrecht nach oben gerichtete (0°) und die diffuse Reflexion (45°) im Wellenlängenbereich 500–850 nm im Labor gemessen. Bei diesen *Labormessungen* reflektierte die Alufolie senkrecht nach oben („gerichtet“) mehr Licht als Lumilys, aber umgekehrt war die diffuse Reflexion (45°) dieser Gewebefolie höher als bei der Alufolie, d. h. dass die Gewebefolie in alle Richtungen reflektierte. Dagegen reflektierte die verschmutzte Alufolie – senkrecht nach oben gerichtet (0°) – zwar ebenfalls weniger, aber bei 45° (diffus) überraschenderweise viel mehr Licht als die saubere Alufolie. Bei beiden Folien lagen die Reflexionsmaxima bei 625–640 nm, ohne dass die Verschmutzung mit braunem Boden die Reflexionsspektren veränderte.

Bei *Feldmessungen* wurde die *Lichtreflexion im sichtbaren Bereich (PAR, 400–700 nm)* im August bei einem Sonnenstandswinkel von 49° in Klein-Altendorf (50°N) mit einem tragbaren Lichtsensor TRP‑3 (Fa. PP-Systems, Amesbury, MA, USA) in 50 cm und 1 m Messhöhe an sonnigen und bewölkten Tagen bestimmt. Bei diesen Feldmessungen reflektierten sowohl die weiße Gewebefolie (Lumilys™) als auch die Alufolie beide überraschenderweise bei leichter bis mittlerer Verschmutzung und beiden Reflexionswinkeln (0° und 45°) am stärksten; die Lichtreflexion nahm erst wieder bei der starken Verschmutzung ab. Beide Folien reflektierten mehr Licht als das Gras der Fahrgasse oder offener Boden im Baumstreifen einer Obstanlage.

Die *Reflexion von UV-B-Strahlen (280–315 nm)* wurde parallel mit einem Optometer X1 (Fa. Gigahertz-Optik, Türkenfels, Deutschland) gemessen, weil sie zusammen mit PAR und kalten Temperaturen die rote Farbbildung bzw. Anthozyansynthese der Fruchtschale fördern. Die senkrecht nach oben gerichtete (0°) UV‑B Reflexion der Alufolie überstieg sowohl an wolkenlosen als auch bedeckten Herbsttagen die der Gewebefolie (Lumilys™). Wie erwartet, nahm die direkte (0°) UV-B-Reflexion von Aluminiumfolie mit der Verschmutzung des Bodens bis zu einem gewissen Grad ab, während die Reflexion von Gewebefolien mit der Verschmutzung des Bodens unerwartet zunahm.

Die Oberflächenrauheit der Reflexionsfolien wurde – in Abhängigkeit vom Verschmutzungsgrad – mit einem Profilometer vom Typ VR-5200 (Fa. Keyence, Osaka, Japan) untersucht. Der aus Falschfarbenbildern abgeleitete Rauheitsindex Sa stieg von 22 µm der sauberen auf 28 µm bei der verschmutzten Gewebefolie Lumilys™, aber von 2 µm bei der sauberen auf 11 µm der verschmutzten Alufolie und erklärte somit den Reflexionsanstieg bei leichter und mittlerer Verschmutzung.

Zusammenfassend lässt sich sagen, dass eine leichte (2–3 g Boden/m^2^) und mittlere (4–12 g Boden/m^2^) Verschmutzung die diffuse Reflexion von PAR (400–700 nm) und UV‑B (280–315 nm) Strahlung der Gewebefolie (Lumilys™) sowie der Alufolie sogar erhöhen kann, sodass z. B. die Gewebefolie auch bei leichter Verschmutzung nochmals verwendet werden kann, während erst die starke Verschmutzung (24–51 g Boden/m^2^) die Lichtreflexion mindert.

## Abstract (en)

The objective of the present work was to study the effects of contamination on the reflective properties of groundcovers used for enhancing fruit colouration in the orchard. Contamination also affects longevity and possible sustainable re-use of materials. A white, woven textile (polypropylene Lumilys™) and silver aluminium foil were experimentally contaminated with soil, similar to the situation after an autumn storm in a fruit orchard. Clean material served as control.

Using a spectrophotometer (StellarNet; Tampa, FL, USA), vertically directed (0°) and diffuse (45°) *light reflection in the range of 500–850 nm* was compared from clean and contaminated groundcover *in the laboratory*. Reflection from vertically directed aluminium foil exceeded that of Lumilys™; however, the highest reflection in all spectral measurements was at 45° (diffuse) from the clean woven textile, i.e., in all directions, and exceeded that of aluminium foil. In contrast, the contaminated vertically directed (0°) aluminium foil reflected less light than the clear foil but, surprisingly, reflected much more light at 45° than the clean foil. Both materials showed reflection peaks at 625–640 nm; light spectra and peaks remained unchanged irrespective of soil contamination.

*Light reflection in the visible range (PAR, 400–700 nm)* was concomitantly measured *in the field* at CKA Klein-Altendorf near Bonn (50°N), Germany, at 0.5 m and 1 m height using a portable TRP‑3 light sensor (PP-Systems, Amesbury, MA, USA) on sunny and cloudy days at a solar angle of 49°. Surprisingly, in these field measurements, Lumilys and aluminium foil reflected most light in both directions (0° and 45°) when slightly to moderately contaminated. Only with heavy contamination did the reflection decrease. Both groundcovers reflected more light than the grass in alleyways of fruit orchards or open soil under the trees.

*UV‑B reflection (280–315 nm)* was examined in parallel in the field using an X1 optometer (Gigahertz Optik, Türkenfels, Deutschland), as it enhances anthocyanin biosynthesis and red fruit colouration in combination with PAR and low temperature. Straight (0°) UV‑B reflection from aluminium foil exceeded that from white woven textile (Lumilys™) on both clear and overcast autumn days. As expected, straight (0°) UV‑B reflection from aluminium foil decreased with soil contamination to a certain extent, but it unexpectedly increased from the woven textile with soil contamination.

Surface roughness in dependence of contamination was measured non-destructively by a profilometer type VR5200 (Keyence, Osaka, Japan). The roughness index, Sa, increased from 22 to 28 µm with soil contamination of the woven textile and from to 2 to 11 µm with aluminium foil, possibly explaining differences in the observed reflectivity.

Overall, the expected severe decline in light reflection (PAR and UV-B) was not seen. In contrast, light (2–3 g soil/m^2^) and moderate (4–12 g soil/m^2^) contamination improved light reflection of PAR (400–700 nm) and UV‑B (280–315 nm) by woven textile (Lumilys™) and aluminum foil. Thus, with slight contamination the materials can be reused, whereas severe contamination (24–51 g soil/m^2^) reduces light reflection.

## Einleitung

Die Ausprägung der roten Frucht- bzw. Deckfarbe bei zweifarbigen Apfelsorten wird am 50° nördlichen Breitengrad erschwert durch den zunehmenden Anbau unter Hagelschutznetzen (Lichtverlust von 8–22 % [Solomakhin und Blanke 2007, 2011]), den Klimawandel mit wärmeren (Nacht‑)Temperaturen sowie durch abnehmende Lichtstärke (Wang et al. 2016), abnehmende Tageslänge und abnehmende Sonnenstandswinkel im Herbst (Saure 1990). Besonders betroffen sind die Standardsorten wie zum Beispiel ‘Gala, Mondial’ und ‘Elstar, Elshof’ und spätreifende Sorten wie ‘Pinova’, ‘Fuji, Kiku 8’, ‘Braeburn’, aber auch ‘Cripps Pink’ mit geringerem genetischen Färbungspotenzial als rote Mutanten (Blanke 2015).

Die Ausprägung der roten Deckfarbe spielt für den Verbraucher bei der Auswahl von Äpfeln und beim Kauf eine wichtige Rolle, da viele Verbraucher die rote Deckfarbe (Anthozyane) mit einem guten Geschmack und Aroma sowie einer optimalen Reife verbinden (King und Cliff 2002). Vielen Inhaltsstoffen – wie zum Beispiel den Anthozyanen und Flavonolen – wird eine positive Wirkung als Antioxidantien im menschlichen Körper zugeschrieben. Diese haben positive Auswirkungen auf Herz‑, Kreislauf- und Krebsleiden (Gallus et al. 2005; Rasmussen et al. 2005). Neben den gesundheitsfördernden Aspekten ist es auch aus betriebswirtschaftlicher Sicht das Ziel, Äpfel mit möglichst guter Fruchtausfärbung zu erzielen und ein Vermarkten in der besseren Handelsklasse 1 oder Extra (Blanke 2015; Schuhknecht et al. 2018).

Zur Verbesserung der Fruchtfärbung gibt es fünf Maßnahmen: 1. reflektierende (Mulch‑)Folien, 2. chemische Farbverstärker, 3. frühzeitige partielle Entlaubung, 4. Sommerriss(-schnitt) und 5. evtl. Verdunstungskühlung – diese Methode erfordert im Spätsommer Zugang zu ausreichend Wasser (Blanke 2015). Reflexionsfolien können die Fruchtausfärbung bzw. den Anteil der roten Deckfarbe und Farbintensität besonders in den inneren und unteren beschatteten Bereichen der Baumkrone verbessern, wo sonst nur eine unzureichende Rotfärbung erzielt wird (Funke und Blanke 2006; Schuhknecht et al. 2018). Weiße reflektierende Gewebefolien wie Lumilys™ (Beaulieu, Belgien) werden in der Mitte der Fahrgassen ausgelegt, um das eingestrahlte Sonnenlicht diffus aus den Fahrgassen in die Baumkrone zu reflektieren (Haaf et al. 2022). Dagegen werden Silberfolien – wie Alufolie bzw. silberbedampfte Polyäthylen-Folien Mylar oder Color-up – direkt unter den Apfelbäumen ausgelegt, um von dort aus das einfallende Sonnenlicht gerichtet nach oben zu reflektieren (Meinhold et al. 2010). Die Ausfärbung der Deckfarbe bzw. Anthozyansynthese wird bestimmt durch die Tag/Nacht-Temperaturdifferenz und die Lichteinstrahlung, sowohl im PAR- (Endt et al. 2002; Takos et al. 2006; Lin-Wang et al. 2011) als auch im UV‑B Bereich (Wu et al. 2012; Xie et al. 2012; Treutter 2010).

**Abb. 1:**
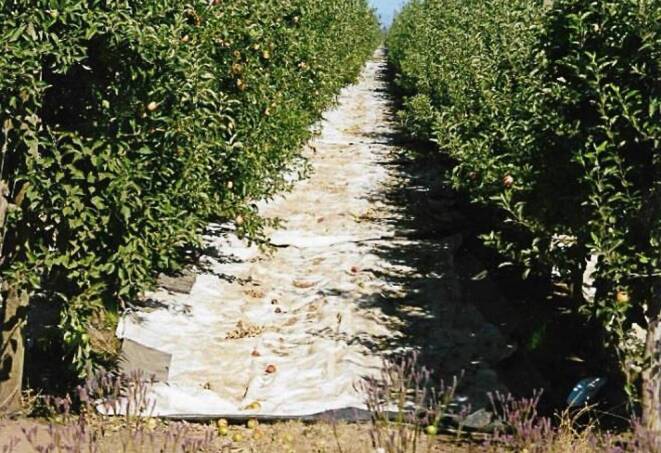
Verschmutzte Reflexionsfolie im Apfelanbau im Herbst in Chile. (© Alvaro Sepúlveda León, Universität Talca, Chile)

In der Obstanlage kann es zu unterschiedlichen Verschmutzungen der Reflexionsfolien kommen – in Form von heruntergefallenen Äpfeln und Blättern sowie Erdstaub, z. B. bei trockenen und warmen klimatischen Bedingungen im Spätsommer und Herbst wie 2018 und 2019. Äste, Äpfel und Blätter lassen sich leicht, staubiger Boden dagegen schwieriger entfernen, wenn er durch Nässe an den Folien haften bleibt und bei Herbststürmen neu angeweht werden kann. Unklar ist, wie sich diese Verschmutzung (Abb. 1) auf die Lichtreflexion von PAR und UV auswirkt. Ziel dieser Arbeit ist es daher, den Einfluss von Verschmutzungen der Reflexionsfolien auf ihre Reflexionseigenschaften zu untersuchen. Dies ist im Hinblick auf die Lebensdauer der Folien im Rahmen einer Strategie zum nachhaltigen (mehrmaligen) Einsatz von Kunststoffen im Gartenbau von Bedeutung.

## Material und Methoden

### Versuchsmaterial

Um die Auswirkungen von Verschmutzungen auf Reflexionsmaterialien zu untersuchen, wurden zwei Folien mit unterschiedlichem Reflexionsverhalten ausgewählt, eine Gewebefolie (Lumilys™), die das Sonnenlicht diffus reflektiert, und eine Alufolie aus 80 % Recyclingmaterial, die das Sonnenlicht gerichtet nach oben reflektiert (Hess und Blanke 2021).

### Weiße Gewebefolie in der Fahrgasse

Bei der weißen Gewebefolie Lumilys WH100 der belgischen Firma Beaulieu Technical Textiles (Kruishoutem, Belgien) handelt es sich um eine leicht glänzende, weiße Gewebefolie mit schmalen rot/grünen Markierungsstreifen. Die Gewebefolie ist mehrfach wiederverwendbar und befahrbar und besitzt laut Herstellerangaben eine Garantie von 7 Jahren und – je nach Beanspruchung – eine Nutzungsdauer von bis zu 10 Jahren. Die Folie ist 2,60 m breit und 100 m lang. Das Material weist ein spezifisches Gewicht von 100 g/m^2^ und eine UV-Garantie von 16.720 MJ/m^2^ auf; die Wasserdurchlässigkeit liegt nach Herstellerangaben bei 0,010 m/s.

### Silberne Alufolie unter den Bäumen

Hierbei wurde eine handelsübliche Aluminiumfolie „Profissimo“ aus dem Drogeriemarkt dm verwendet, da sie zu 80 % aus recyceltem Aluminium hergestellt wird. Ihre hohe Reißfestigkeit wird durch eine Prägungsstruktur erzielt. Die Folie ist auf der einen Seite glänzend und auf der Rückseite matt. Sie eignet sich – wie Mylar und Color-up – in der Regel zum einmaligen Auslegen.

### Verschmutzungen

**Abb. 2:**
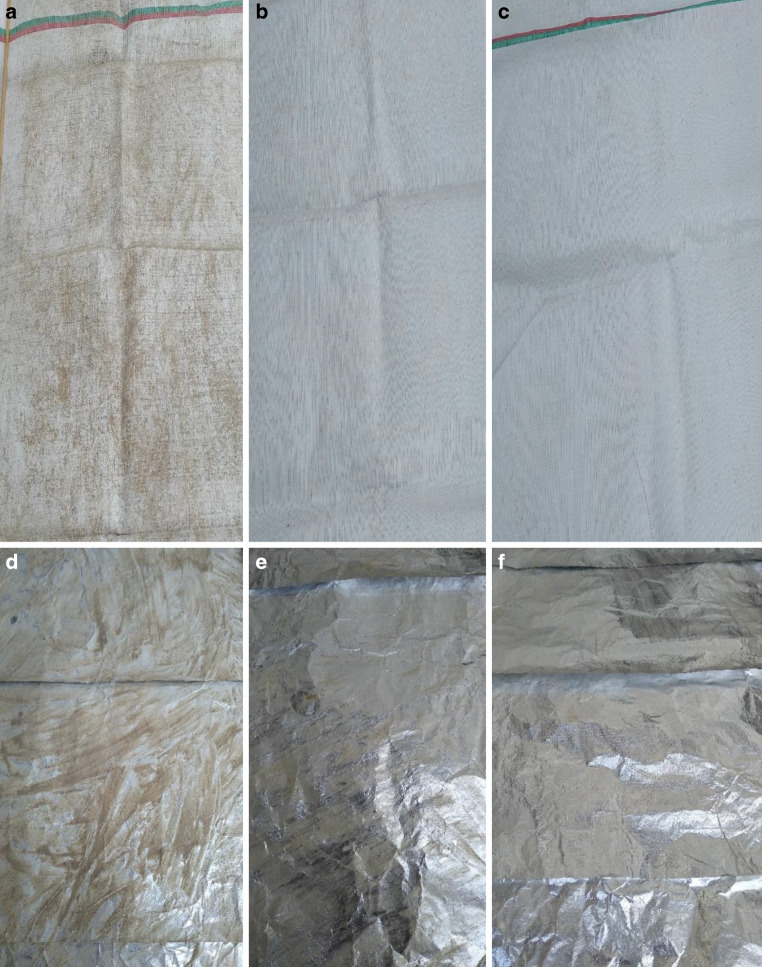
Verschmutzte Gewebefolie (**a**–**c**) und verschmutzte Alufolie (**d**–**f**), **a**,**d** starke Verschmutzung, **b**,**e** mittlere Verschmutzung, **c**,**f** leichte Verschmutzung

Die Verschmutzung der beiden Reflexionsfolien erfolgte mit Boden aus dem Ap Horizont (Braunerde). Von jeder Folie wurden drei gleich große Stücke (1,00 m x 1,20 m) unterschiedlich stark verschmutzt (Tab. 1), um die Abstufung leichte, mittlere und starke Verschmutzung zu erzielen (Abb. 2). Das vierte Folienstück wurde nicht verschmutzt und diente als Kontrolle (Tab. 1). Aufgrund der glatten Oberfläche der Alufolie und Lumilys wurde der Boden nach dem Auftragen mit Wasser leicht angefeuchtet, um eine bessere Haftung des Bodens auf der Folie zu erzielen; gemessen wurde die Lichtreflexion der Folien nach dem Antrocknen.

**Tab. 1:** Verschmutzungsgrade der beiden Reflexionsfolien Lumilys und Alufolie

In g Boden/m2	Lumilys	Alufolie
*Sauber (Kontrolle)*	0	0
*Leichte Verschmutzung*	3	2
*Mittlere Verschmutzung*	12	4
*Starke Verschmutzung*	51	24

### Messungen der Reflexionsspektren im Labor

**Abb. 3:**
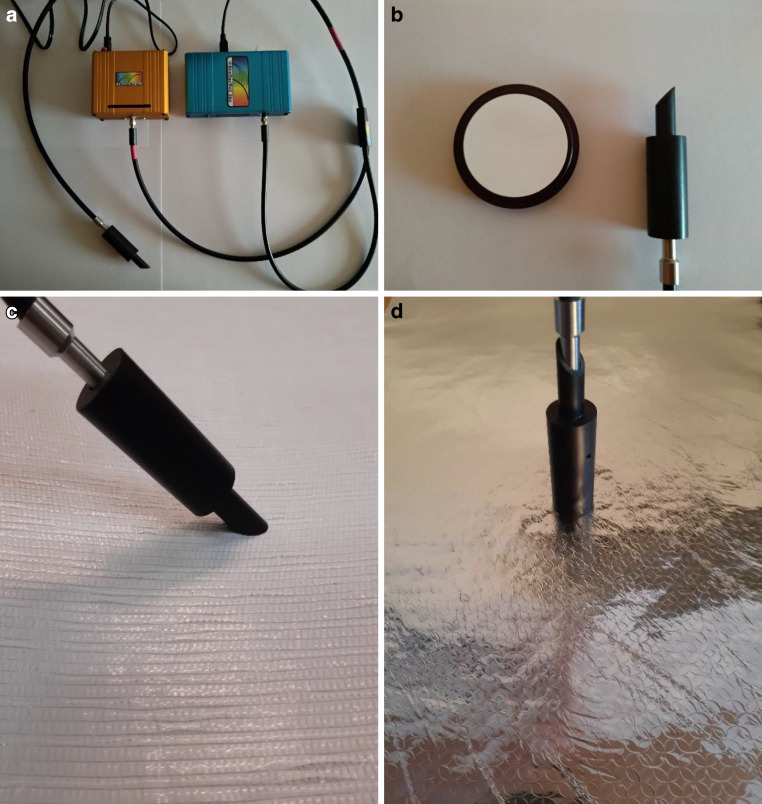
**a** Lichtquelle (*gelb*) und BLUE-WAVE-Box (*blau*) angeschlossen an eine Glasfaser. **b** Gepresstes Bariumsulfatpulver zum Kalibrieren bzw. Weißabgleich (100 % Reflexion) mit Messkopf für 45° Reflexionsmessungen (Eingangswinkel = Abgangswinkel); **c** Messung der Lumilys Kontrolle im 45° Reflexionswinkel; **d** Messung der sauberen Alufolie (Kontrolle) im 0° (senkrecht) Reflexionswinkel

Die spektrale Messung der Lichtreflexion erfolgte mit einem Spektrometer der Firma StellarNet (Florida, USA) im Labor bei einem Winkel von 0° und 45° (Einfallswinkel = Ausfallswinkel). Zu dem Messgerät gehört eine Lichtquelle (12 V, SL1 Wolframhalogenlampe mit einem Spektralbereich von 350–2500 nm), die BLUE-WAVE-Box und der Lichtwellenleiter „Blue Wave“ (200–1150 nm), der das Licht mittels Glasfaser (R600-8-VIS-NIR) zur Messsonde (Cosinus korrigiert) leitet (Abb. 3a,b).

Vor jeder Messreihe mit dem empfohlenen Messeinstellungen (Tab. 2) wurde ein Weißabgleich bei dem jeweiligen Winkel (0° oder 45°) (Abb. 3b) mithilfe eines mitgelieferten Reflexionsstandards RS50 (gepresstes Bariumsulfatpulver) mit 50 mm Durchmesser durchgeführt, um das Spektrometer auf 100 % Reflexion zu kalibrieren und die Messwerte diffuser Reflexion von der Gewebefolie (Abb. 3c) und gerichteter Reflexion von der Alufolie (Abb. 3d) mit der ebenfalls mitgelieferten Software „Spectra Wiz Shortcut“ ausgewertet.

**Tab. 2:** Standardeinstellungen des Spektrometers (StellarNet)

Einstellungen	Standardeinstellungen
Optische Auflösung	0,2–0,6 nm (abhängig von der Wellenlänge)
Temperaturkompensation	1 (ein)
Anzahl der Scans	5
Integrationszeit	50 ms
Messabstand	0 cm

### Messungen der PAR-Lichtreflexion (400–700 nm) im Feld

**Abb. 4:**
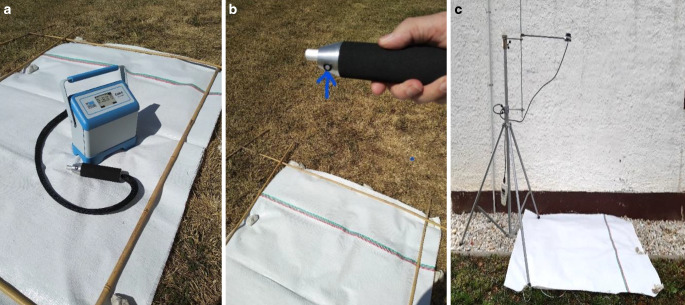
**a** EGM‑5 mit Lichtsensor TRP‑3 auf der Lumilys Gewebefolie, **b** mit *blauem* *Hinweispfeil* auf den Messkopf des TRP‑3, **c** feststehendes Messstativ (zur besseren Darstellung und Erkennung vor einer Hauswand), sodass nur die Folien ausgetauscht bzw. bewegt werden und der Sensor in seiner Position verbleibt. Der Sonnenstandswinkel lag am 21.08.2019, am 50° nördlichen Breitengrad, bei 49° und die PAR-Einstrahlung in der Sonne bei 1580 µmol/m^2^/s und im Baumschatten bei 276 µmol/m^2^/s

Die Lichtreflexion im sichtbaren Bereich (PAR 400–700 nm) wurde an einem klaren Sommertag ohne Wolken (21.08.2019) von 13 Uhr bis 14.30 Uhr, auf dem Campus Klein-Altendorf (50°N) der Universität Bonn, mit einem tragbaren EGM‑5 mit einem Cosinus korrigierten TRP‑3 Lichtsensor (Fa. PP-Systems, Amesbury, MA, USA) gemessen. Das EGM‑5 eignet sich für Feldmessungen der Lufttemperatur (0–50 °C), Luftfeuchte und PAR (400–700 nm) im Bereich 0–3000 µmol/m^2^/s. Die Untersuchungen erfolgten bei einem Ausfallswinkel von 0° und 45° der reflektierten Sonnenstrahlen über den Reflexionsfolien und über Gras (Abb. 4a–c).

**Abb. 5:**
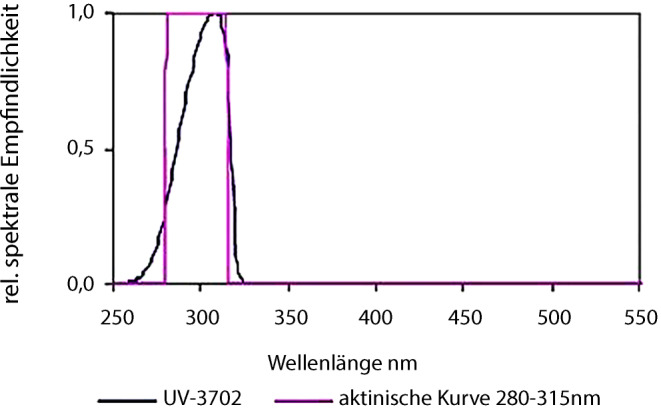
Spektrale Empfindlichkeit des Optometers (Gigahertz-Optik, Türkenfeld, Deutschland) mit Empfindlichkeitsmaximum um ca. 310 nm

Alle Feldmessungen (PAR und UV-B) wurden nach internationalem Standard in 1 m Höhe und 50 cm über dem Boden mit einem Messstativ durchgeführt (Abb. 5c). Zur Bestimmung der Lichtreflexion wurde der Lichtsensor im 45°-Winkel (schräg nach unten) und 0°-Winkel (senkrecht nach unten) über die Reflexionsfolien montiert. Bei allen Reflexionsmessungen handelte es sich um die gleichen verschmutzten Folienstücke, die auch bei der spektralen Messung und den Messungen mit dem 3D-Profilometer verwendet wurden. Für jeden Verschmutzungsgrad, d. h. keine, leichte, mittlere und starke Verschmutzung, wurden 20 Messungen durchgeführt.

### Messungen der UV-B Reflexion im Feld

Die UV‑B Reflexion (280–315 nm) wurde mit einem tragbaren Optometer X1 und einem Cosinus korrigiertem UV‑B Detektor (UV-3702) der Firma Gigahertz-Optik (Türkenfeld, Deutschland) von 0° (vertikal) gemessen (Tab. 3, Abb. 5).

**Tab. 3:** Detektor Spezifikationen (Gigahertz-Optik)

UV‑B Detektor (UV-3702)	Spezifikationen
Spektrale Empfindlichkeit	UV‑B 280–315 nm
Empfindlichkeit	1,7 nA/(W/m^2^)
Max. Empfindlichkeit	Ca. 310 nm (geschätzt)
Eingangsoptik	Cosinus Blickfeld
Eingangsoptik	11 mm ⌀ Diffusorfenster

Die UV‑B Messungen wurden an einem wolkenlosen Tag (21.08.2019) – wie die PAR-Messungen – und zusätzlich an einem Tag mit diffuser Einstrahlung (08.08.2019) durchgeführt. Die UV-B-Einstrahlung, im 0°-Winkel vertikal nach oben, am 21.08.2019 betrug zur Zeit der Messung in der Sonne 802 mW/m^2^ und im Baumschatten 409 mW/m^2^ und am 08.08.2019 unter diffusen Lichtbedingungen 113 mW/m^2^.

## Rauheitsmessungen und Rauhigkeitsparameter

Der Rauheitsgrad sauberer und verschmutzter Alu- und Textilfolie wurde mit einem 3D-Profilometer vom Typ VR 5200 (Keyence Osaka, Japan) bei 40facher Vergrößerung in 200 × 100 mm Abschnitten bestimmt und der Rauhigkeitsgrad Sa (Arithmetische mittlere Höhe) nach folgender Formel berechnet:

1 $$Sa=\frac{1}{A}{\iint }_{A}^{k}\left| Z\left(x{,}y\right)\right| dxdy$$

Der Parameter Sa ist die mittlere arithmetische Differenz zwischen Erhebungen und Vertiefungen des gemessenen Profils, wobei S für das flächige Rauheitsprofil steht; Sa ist der Durchschnitt (a = average) der absoluten Werte und A = area/Fläche (250 × 100 mm). Das Messobjekt wird dabei mit rotem, grünem und blauem Licht beleuchtet. Die Auswertung erfolgt nach Herstellerangaben über Messalgorithmen – mit dem sogenannten TMT Algorithmus (Telecentric Multi-Triangulation) einer telezentrischen Mehrfachtriangulation.

### Statistische Auswertungen

Die Daten der Feldmessungen wurden einer einfaktoriellen Varianzanalyse (ANOVA) mithilfe von SPSS Version 26 (IBM, New York, USA) unterzogen. Die Daten wurden mittels des Kolmogorov-Smirnov-Tests auf Normalverteilung geprüft. Als Post hoc wurde der Bonferroni-Test durchgeführt, wenn es zu signifikanten Unterschieden zwischen mehreren Gruppen kam.

## Ergebnisse

### Spektrale Lichtreflexion der weißen Gewebefolie (Lumilys)

**Abb. 6:**
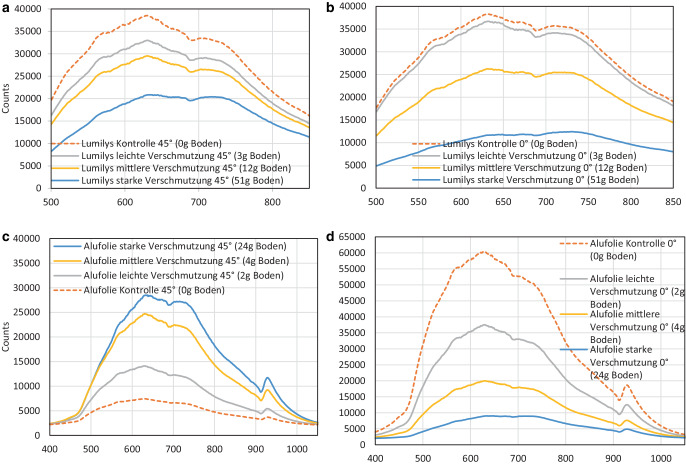
Einfluss der Verschmutzung auf die Lichtreflexionsspektren der weißen Gewebefolie (Lumilys) **a** *diffus *(45°) (oben links) **b** senkrecht nach oben* gerichtet *(0°,) sowie **c** der Alufolie *diffus *(45°) und **d** *Alufolie *senkrecht nach oben* gerichtet *(0°) (*n* = 12 Messungen pro Verschmutzungsgrad) in relativen Einheiten (counts)

Die stärkste *diffuse Lichtreflexion* wurde bei der weißen Gewebefolie ohne Verschmutzung bei 625–640 nm mit 38.504 Counts erreicht. Mit steigender Verschmutzungsintensität verschlechterten sich die Reflexionseigenschaften. Das untere Maximum bei 20.862 Counts wurde – wie erwartet – bei der weißen Gewebefolie (Lumilys) mit starker Verschmutzung von 51 g Boden pro m^2^ gemessen (Abb. 6a). Bei dem Kurvenverlauf konnten keine Unterschiede zwischen den Verschmutzungsgraden festgestellt werden.

Bei der senkrecht nach oben* gerichteten Reflexion* (0°) der weißen Gewebefolie (Lumilys) in Abb. 6b war der Kurvenverlauf fast identisch zu dem in Abb. 6a. Die Lumilys Kontrolle ohne Verschmutzung hatte ihr Reflexionsmaximum bei 625–640 nm mit 38.318 Counts, also etwas geringer als bei der Lumilys Kontrolle im 45°-Reflexionswinkel. Das untere Maximum wurde wiederum bei der Lumilys mit der starken Verschmutzung mit nur 12.433 Counts bei ca. 725 nm gemessen. Das war jedoch mit 8428 Counts deutlich geringer als bei der weißen Gewebefolie (Lumilys) mit der starken Verschmutzung im 45° Reflexionswinkel. Die weiße Gewebefolie (Lumilys) ohne Verschmutzung erreichte bei der gerichteten Reflexion im 0°-Winkel ein Maximum von ca. 91 % bei 700 nm und das Minimum wurde wiederum bei der Lumilys mit der starken Verschmutzung erreicht, wo die Reflexion nur noch bei ca. 27 % bei einer Wellenlänge von 700 nm lag (Abb. 6b).

### Spektrale Lichtreflexion der Alufolie

Die **saubere Alufolie **(Kontrolle) war die große Ausnahme bzw. Überraschung: Sie **wies die geringste** *diffuse Lichtreflexion* (45°-Reflexionswinkel) mit einem Maximum von 7444 Counts bei 625–640 nm auf und mit 28.511 Counts **die höchste Lichtreflexion bei der starken Verschmutzung** (24 g Boden). Der Kurvenverlauf war ebenfalls fast identisch zu denen aus Abb. 6a,b. Die Alufolie mit der starken Verschmutzung reflektierte bei 700 nm ca. 64 % des eingestrahlten Lichtes (Abb. 6c), wohingegen die saubere Alufolie bei 700 nm nur noch ca. 18 % des eingefallenen Lichtes reflektierte (Abb. 6c).

Abb. 6d stellt die senkrecht nach oben* gerichtete Reflexion* (0°) der Alufolie in Counts dar: je stärker die Verschmutzung, umso geringer ist die Lichtreflexion. Die saubere Alufolie (Kontrolle) erreichte ein Maximum von 60.488 Counts bei 625–640 nm, d. h. die gleichen Wellenlängen wie bei Lumilys (Abb. 6b). Das untere Maximum lag ebenfalls bei 625–640 nm mit 9045 Counts bei der stark verschmutzten Alufolie mit 24 g Boden pro m^2^.

### Reflexionsspektren von Lumilys und Alufolie im Vergleich zu Gras und Boden

**Abb. 7:**
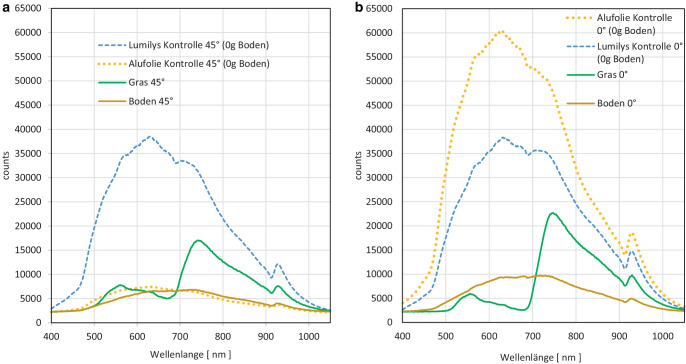
Spektren der **a** diffusen (45°-Reflexionswinkel) und **b** der senkrecht nach oben gerichteten Reflexion im 0°-Reflexionswinkel jeweils der sauberen Alufolie (*gelb*), der sauberen Gewebefolie (*blau*) sowie von Gras in der Fahrgasse (*grün*) und Boden im Baumstreifen (*braun*) in rel. Einheiten (counts; *n* = 12 Messungen pro Verschmutzungsgrad)

Die *diffuse Reflexion* (45°) der sauberen weißen Gewebefolie ohne Verschmutzung (Lumilys Kontrolle) wies bei ca. 630 nm mit 38.504 Counts den höchsten Reflexionswert auf. Die saubere Alufolie ohne Verschmutzung (Alu Kontrolle) reflektierte nur unwesentlich mehr als unbewachsener Boden. Bei der diffusen Reflexion von **Gras** betrug das erste Maximum bei ca. 564 nm ca. 7782 Counts und das zweite und höhere Reflexionsmaximum betrug bei ca. 750 nm ca. 16.915 Counts (Abb. 7a).

Bei der Bestimmung der senkrecht nach oben *gerichteten Reflexion *(0°) drehte sich die Reihenfolge der Reflexionsmaxima um: Die Alufolie ohne Verschmutzung (Alu Kontrolle) reflektierte am stärksten bei ca. 630 nm mit 60.488 Counts, gefolgt von einem Maximum von 38.318 Counts bei ca. 630 nm von der sauberen weißen Gewebefolie (Lumilys) ohne Verschmutzung (Kontrolle). **Boden und Grasaufwuchs zeigten mit ca. 5000 mehr Counts bei ca. 750** **nm eine um ca. 25** **% höhere gerichtete als diffuse Reflexion** (Abb. 7a,b).

Die Reflexionsmaxima der Folien lagen bei 625–640 nm (Abb. 6a–c). Die senkrecht nach oben gerichtete Reflexion (0°) der sauberen Alufolie war stärker als bei der sauberen Lumilys, aber die diffuse Reflexion (45°) der sauberen Lumilys höher als bei der sauberen Alufolie (Abb. 7a,b). Bei der Lumilys blieben die Reflexionsmaxima bei Veränderung des Messwinkels in der gleichen Größenordnung und bei den gleichen Wellenlängen, d. h. die weiße Gewebefolie reflektiert das Licht diffus gleichmäßig in alle Richtungen. Dagegen reflektierte die Alufolie wesentlich stärker im senkrechten 0°-Winkel als im 45°-Winkel, was auf ihre gerichtete Reflexion hinweist.

### Feldmessung der PAR-Reflexion der weißen Gewebefolie bei klarem Himmel und Sonne

**Abb. 8:**
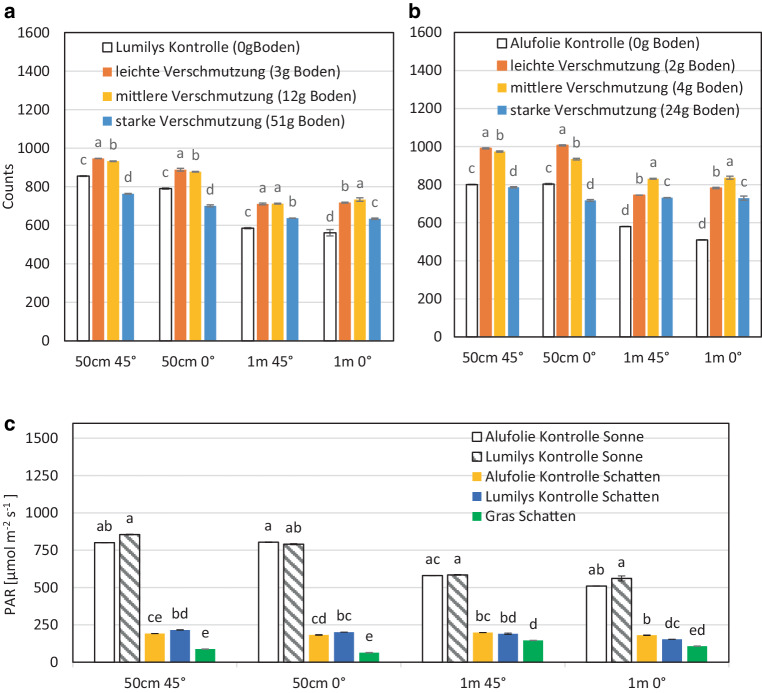
Diffuse (45°) und gerichtete (0°) Reflexion (PAR in µmol/m^2^/s) **a** der weißen Gewebefolie (Lumilys), und **b** der Alufolie 50 cm und 1 m über den Folien, Sonnenstandswinkel 49° in Klein-Altendorf am 21.08.2019, klar ohne Wolken, und **c** diffuse (45°) und gerichtete (0°) Reflexion (PAR) der sauberen Alufolie und der sauberen weißen Gewebefolie in der Sonne und im Schatten sowie des Grases im Schatten, 50 cm und 1 m über den Folien bzw. Grases, (*n* = 20 Messungen). Unterschiedliche Buchstaben a, b, c, d bedeuten signifikante Unterschiede bei *p* ≤ 0,05 in den Gruppen (50 cm 45°, 50 cm 0°, 1 m 45°, 1 m 0°)

Neben den spektralen Labormessungen (Abb. 4, 5, 6 und 7) wurde die Reflexion im sonnig-heißen August 2019 in der Obstanlage Klein-Altendorf gemessen. Dabei erhöhte eine leichte und mittlere Verschmutzung überraschenderweise die Reflexion der weißen Gewebefolie Lumilys, sank aber bei der starken Verschmutzung. Dies galt sowohl bei 0° als auch 45° Reflexionswinkel bei 50 cm und 100 cm Abstand von der Folie (Abb. 8a).

Die diffuse Reflexion (45°) der sauberen weißen Gewebefolie erreichte bei 50 cm bzw. 1 m ca. 855 µmol/m^2^/s bzw. 585 µmol/m^2^/s sowie die gerichtete Reflexion (0°) ca. 791 µmol/m^2^/s (50 cm) bzw. 561 µmol/m^2^/s (1 m). Bei beiden Messhöhen führte eine leichte und mittlere Verschmutzung nicht zu einer Abnahme, sondern überraschenderweise zu einer Erhöhung der Lichtreflexion und eine starke Verschmutzung wiederum zu einem Absinken sowohl der diffusen als auch der gerichteten Reflexion (Abb. 8a). In Tab. 4 wurden die Messwinkel 0° (senkrecht) und 45° bei 1 m und 50 cm Messhöhe miteinander verglichen, um das Verhältnis zwischen diffuser und gerichteter Reflexion zu ermitteln und zu sehen, ob eine Verschmutzung den Anteil diffuser oder gerichteter Reflexion verändert bzw. erhöht.

**Tab. 4:** Verhältnis zwischen diffuser (45°) und gerichteter (0°) Reflexion (PAR) der weißen Gewebefolie Lumilys und der Alufolie in 1 m und 50 cm Höhe (in µmol – nach Abb. 8a)

*Messabstand*	*Verhältnis*	*Lumilys sauber*	*Lumilys leichte Verschmutzung*	*Lumilys mittlere Verschmutzung*	*Lumilys starke Verschmutzung*
1 m	0°:45°	561: 585	718: 711	734: 712	634: 637
50 cm	0°:45°	791: **855**	889: **947**	878: **933**	701: **764**
*Messabstand*	*Verhältnis*	*Alufolie sauber*	*Alufolie leichte Verschmutzung*	*Alufolie mittlere Verschmutzung*	*Alufolie starke Verschmutzung*
1 m	0°:45°	510: 580	782: 745	837: 831	729: 732
50 cm	0°:45°	**804:** 801	**1006**: 991	934: **974**	717: **786**

Bei dem weiteren Messabstand (1 m) war die diffuse (585 µmol/m^2^/s) und gerichtete (561 µmol/m^2^/s) Reflexion der sauberen Lumilys ähnlich, aber bei geringerer Messhöhe (50 cm) war die diffuse Reflexion um 55–63 µmol/m^2^/s mit ca. 6 % etwas höher als die gerichtete (0°) Reflexion.

## PAR-Reflexion der Alufolie (Feldmessung)

Die Alufolie reflektierte bei 45° und 0° mit den höchsten Reflexionswerten bei leichter und mittlerer Verschmutzung. Bei 50 cm Abstand im 45°-Winkel lag der höchste Reflexionswert bei der leichten Verschmutzung (2 g Boden) mit 991 µmol/m^2^/s, gefolgt von der mittleren Verschmutzung (4 g Boden) mit 974 µmol/m^2^/s, der Kontrolle mit 800 µmol/m^2^/s und der starken Verschmutzung (24 g Boden) mit 786 µmol PAR/m^2^/s. Bei 50 cm Abstand im 0°-Winkel wurde mit 1006 µmol PAR/m^2^/s der größte Wert für Alufolie ebenfalls bei der leichten Verschmutzung gemessen, gefolgt von der mittleren Verschmutzung mit 934 µmol/m^2^/s und der Kontrolle (saubere Alufolie) mit 804 µmol/m^2^/s. Die tiefsten Reflexionswerte wurden wieder bei der starken Verschmutzung mit 717 µmol/m^2^/s gemessen, bei der die ganze Fläche mit Schmutz bedeckt war (Abb. 2d). In 1 m Abstand über der Alufolie im 45°-Winkel war die höchste diffuse Reflexion bei mittlerer Verschmutzung 831 µmol/m^2^/s, gefolgt von der leichten Verschmutzung mit 745 µmol/m^2^/s und der starken Verschmutzung mit 732 µmol/m^2^/s. Die Alufolie ohne Verschmutzung reflektierte mit 580 µmol/m^2^/s, d. h. 151 µmol/m^2^/s weniger Licht als bei starker Verschmutzung. Bei 1 m und senkrechtem (0°) Reflexionswinkel wurde der größte Wert ebenfalls bei der mittleren Verschmutzung mit 837 µmol/m^2^/s gemessen, dicht gefolgt von der leichten Verschmutzung mit 782 µmol/m^2^/s. Bei der starken Verschmutzung lag der Mittelwert bei 729 µmol/m^2^/s und bei der Kontrolle der geringste Mittelwert mit 510 µmol/m^2^/s (Tab. 4).

Bei der leichten und mittleren Verschmutzung reflektierte die Alufolie bei beiden Winkeln (0° und 45°) und bei beiden Messhöhen (50 cm und 1 m) unerwartet mehr Licht als die unverschmutzte Kontrolle (Abb. 8b).

## PAR-Reflexion von Lumilys, Alufolie im Vergleich zu Gras/Boden (Feldmessung)

Beide Mulchfolien (Lumilys und Alufolie) reflektierten bei 50 cm Abstand über dem Boden etwa das 4Fache als frisch gemähtes Gras in der Fahrgasse; dieser Wert reduzierte sich auf das 3Fache bei 1 m Messabstand. Auch im Schatten reflektierten sowohl die Mulchfolien, im Herbst bei niedrigem Sonnenstandswinkel, als auch das Gras noch einen erheblichen Teil des sichtbaren Lichtes (PAR) (Abb. 8c).

### Gerichtete UV-B Reflexion der weißen Gewebefolie – Feldmessung an sonnigen Tagen

**Abb. 9:**
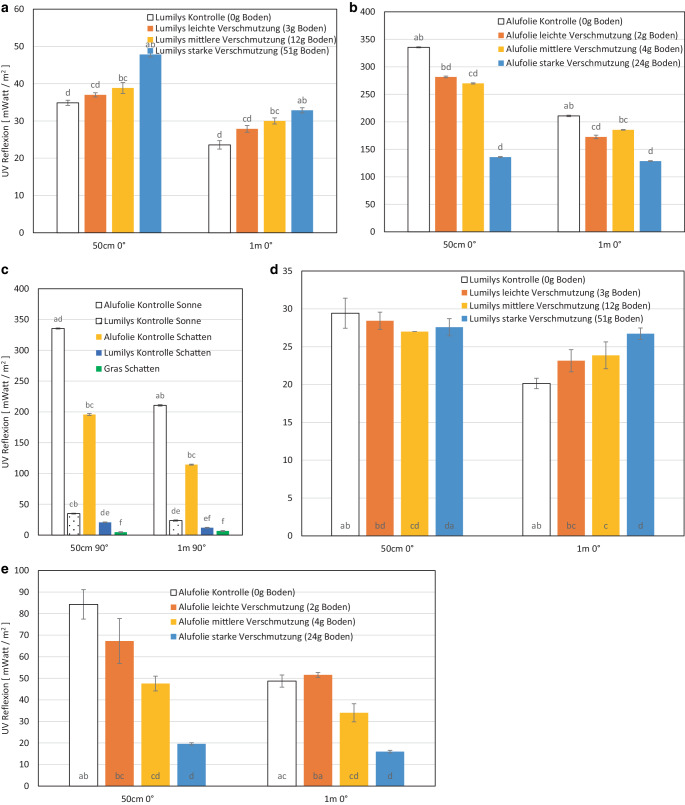
*Gerichtete UV‑B Reflexion (*in mW/m^2)^ **a** *der weißen Gewebefolie* (Lumilys) und **b** der Alufolie, jeweils 50 cm und 1 m über den Folien, Sonnenstandswinkel 49°, am 21.08.2019 am CKA Klein-Altendorf, Sonnentag ohne Wolken. sowie **c** *gerichtete UV‑B Reflexion* (in mW/m^2^) der sauberen Alu- und sauberen Gewebefolie Lumilys in der Sonne und im Schatten sowie Gras im Schatten im 50 cm und 1 m über den Folien am gleichen Tag sowie **d** *Gerichtete UV‑B Reflexion* (0°) (in mW/m^2^) der weißen Gewebefolie (Lumilys) und **e** der Alufolie 50 cm und 1 m über den Folien, Sonnenstandswinkel 53° am 08.08.2019, wolkig (diffus). (*n* = 7 Messungen pro Verschmutzungsgrad; unterschiedliche Buchstaben bedeuten signifikante Unterschiede bei *p* ≤ 0,05 in den beiden Gruppen 50 cm bzw. 1 m; UV‑B Einstrahlung 113 mW/m^2^)

Die *gerichtete UV‑B Reflexion* (0°) der sauberen weißen Gewebefolie (Lumilys) war mit 35 mW/m^2^ in 50 cm über der Folie an einem sonnigen Tag ohne Wolken am geringsten (Abb. 9a). Mit steigendem Verschmutzungsgrad reflektierte die Lumilys nicht weniger, sondern unerwartet mehr UV‑B. So wurde die stärkste UV‑B Reflexion (280–315 nm) mit ca. 48 mW/m^2^ bei der starken Verschmutzung mit 51 g Boden pro m^2^ gemessen. Die mittlere Verschmutzung mit 12 g Boden erzielte mit 39 mW/m^2^ und die leichte Verschmutzung mit 3 g Boden mit 37 mW/m^2^ mittlere Reflexionswerte, genauso wie die Messungen in 1 m über den Folien (Abb. 9a).

## Gerichtete UV-B Reflexion der Alufolie – Feldmessung an sonnigen Herbsttagen

Die senkrecht nach oben *gerichtete (0°) UV‑B Reflexion*** der sauberen Alufolie** war mit ca. 335 mW/m^2^ in 50 cm bzw. 210 mW/m^2^ in 1 m Höhe am gleichen sonnigen Tag am stärksten und **nahm mit steigendem Verschmutzungsgrad ab** – im Gegensatz zur Gewebefolie Lumilys (Abb. 9a). Die Alufolie mit der leichten Verschmutzung reflektierte in 50 cm (bzw. 1 m Höhe) ca. 282 (173) mW/m^2^, bei der mittleren Verschmutzung ca. 270 (185) mW/m^2^ und bei der starken Verschmutzung ca. 136 mW/m^2^ an UV‑B Strahlung.

### Gerichtete UV-B Reflexion von Lumilys und Alufolie im Vergleich zu Gras und Boden (Feldmessung)

Die senkrecht nach oben gerichtete (0°) UV‑B Reflexion der sauberen Alufolie verdoppelte sich von der Messung im Schatten mit ca. 196 mW/m^2^ auf ca. 335 mW/m^2^ in der Sonne in 50 cm Abstand (Abb. 9c); die gleichen Verhältnisse traten in 1 m Abstand auf (130 mW/m^2^ im Schatten bzw. 210 mW/m^2^ in der Sonne). Mit 196 mW/m^2^ reflektierte die saubere Alufolie durch ihre vorzugsweise gerichtete Reflexionseigenschaft im Schatten in 50 cm etwa das 6Fache der sauberen Gewebefolie (Lumilys) mit 35 mW/m^2^. Im Schatten war die gerichtete (0°) UV‑B Reflexion beider Folien mit ca. 196 mW/m^2^ bzw. 21 mW/m^2^ wesentlich höher als Gras mit ca. 5 mW/m^2^; ähnliche Verhältnisse traten in 1 m Abstand auf (Abb. 9c).

### Gerichtete UV-B Reflexion der weißen Gewebefolie bei diffuser Einstrahlung – Feldmessung bei bewölktem Himmel

Am 50. Breitengrad sind Herbsttage häufig von diffuser Einstrahlung geprägt. Die gerichtete UV‑B Reflexion (0°) der sauberen und leicht verschmutzten weißen Gewebefolie (Lumilys) in 50 cm war mit ca. 29 mW/m^2^ bei diffuser Einstrahlung am stärksten, gefolgt von der mittleren und starken Verschmutzung mit ca. 27 mW/m^2^. Überraschenderweise drehte sich die Reihenfolge der Reflexionswerte in 1 m Abstand um (Abb. 9d) – wie bei der Reflexion der gleichen Gewebefolie mit vorwiegend diffuser Reflexion an einem sonnigen Tag (Abb. 9a).

### Gerichte UV-B Reflexion der Alufolie bei diffuser Einstrahlung – Feldmessung bei bewölktem Himmel

Die gerichtete UV‑B Reflexion (0°) der Alufolien nahm mit zunehmender Verschmutzung in einer Höhe von 50 cm über den Folien von ca. 84 mW/m^2^ auf ca. 20 mW/m^2^ ab (Abb. 9e) – ähnlich wie bei den Gewebefolien (Abb. 9d) und wiederholte sich in 1 m.

## Oberflächenrauhigkeit

**Abb. 10:**
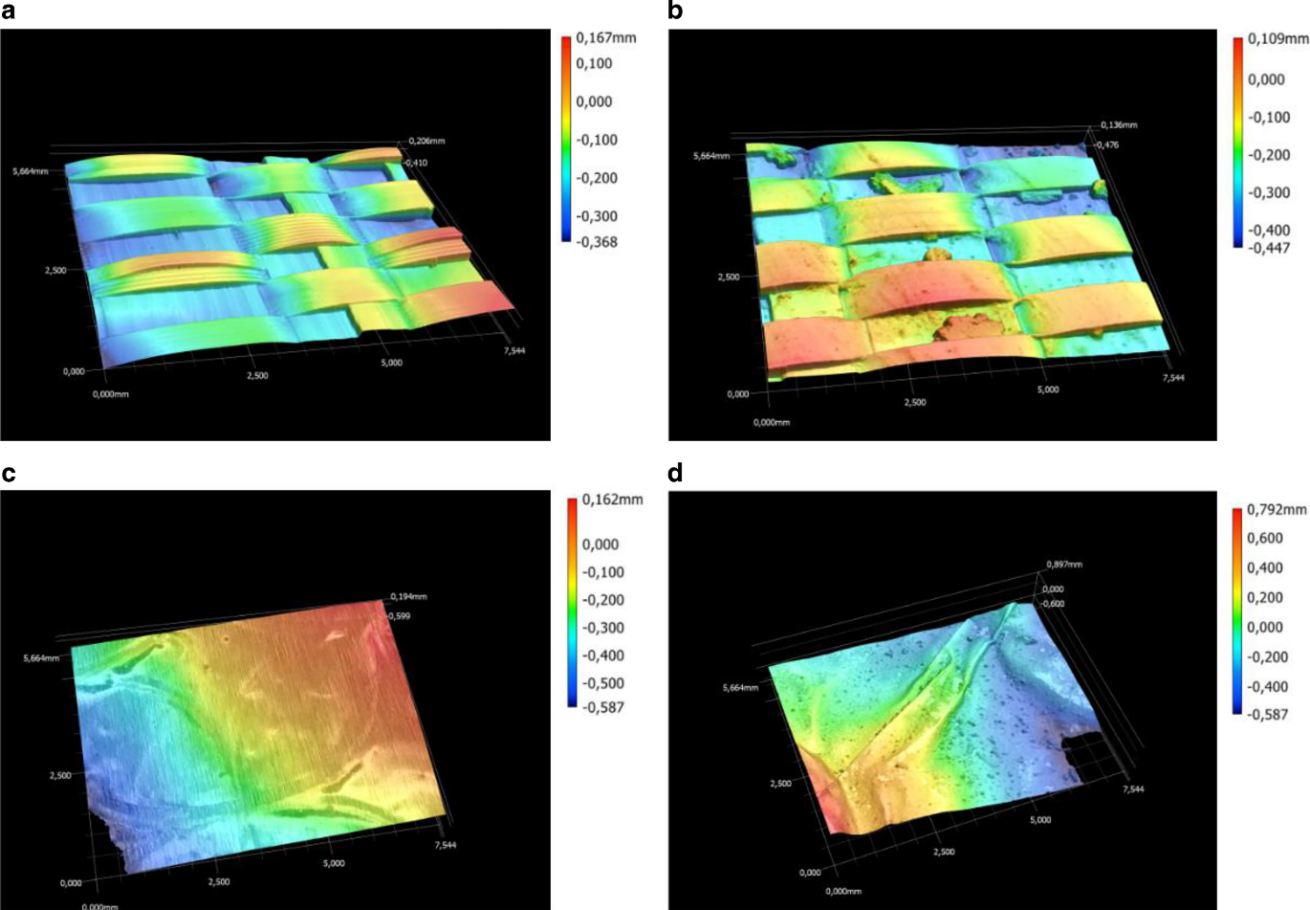
Falschfarbenbilder der sauberen (**a**) und verschmutzten (**b**) weißen Gewebefolie Lumilys sowie der sauberen (**c**) und verschmutzten Alufolie (**d**); Vertiefungen in *blau* und Erhebungen in *rot*; 40fache Vergrößerung

Besonders der Anstieg der diffusen (45°) Lichtreflexion der Alufolie trotz stärkerer Verschmutzung (Abb. 6c und 8b) lässt sich erklären, wenn man die Oberflächen unter einem Mikroskop betrachtet. Die Falschfarbendarstellung, d. h. Vertiefungen in blau und Erhöhungen in rot, zeigt die zunehmende Oberflächenrauhigkeit mit zunehmender Verschmutzung (Abb. 10). Der Rauhigkeitsindex Sa stieg mit zunehmender Verschmutzung bei der Gewebefolie Lumilys von ca. 22 µm auf 28 µm und der Alufolie von ca. 2 auf 11 µm (Tab. 5).

**Tab. 5:** Rauhigkeitsindex Sa der weißen Gewebefolie und Alufolie (Minimum, Maximum, Mittelwert, in µm)

Sa	Minimum	Maximum	Mittelwert
*Weiße Gewebefolie*			
Sauber	20,9	23,9	21,9
Verschmutzt	28,0	28,0	28,0
*Alufolie*			
Sauber	1,87	2,02	1,95
Verschmutzt	10,9	11,1	11,0

## Diskussion

Da die rote Deckfarbe der Äpfel eine Reaktion auf die abiotischen Umwelteinflüsse ist, ist die Merkmalsausprägung von Jahr zu Jahr unterschiedlich. Die Fruchtfarbe ist jedoch der Hauptanreiz für die Konsumenten, die Äpfel zu kaufen (Bosančić et al. 2018). In Jahren mit schlechter Farbausprägung können Reflexionsfolien die Ausprägung der roten Deckfarbe verstärken und dadurch eine möglichst gleichbleibende hohe Qualität der Früchte sichern (Blanke 2015). Durch die verbesserte Ausprägung der roten Deckfarbe bei den Äpfeln wurden ein höherer Anteil an Klasse 1 und somit auch höhere finanzielle Erlöse erzielt (Blanke 2015). Die reflektierende Gewebefolie Extenday erreichte den größten Anteil (69 %) am besten gefärbter Früchte (75–100 % Fruchtfärbung), gefolgt von der weißen Gewebefolie (Lumilys) mit einem Anteil von 44 % im Vergleich zur Kontrolle mit Gras. Reflexionsfolien können an einem bewölkten Herbsttag am 50° N, wenn das Licht besonders für die Rotfärbung gebraucht wird, bis zu 29 % mehr Licht (PAR) reflektieren als das Gras in der Fahrgasse (Solomakhin und Blanke 2007).

### Sonnenstandswinkel

Der Sonnenstandswinkel ist maßgeblich an der Rotfärbung der Äpfel beteiligt. Am 50° nördlichen Breitengrad nimmt der maximale Sonnenstandswinkel rapide von 62° um 13 Uhr im Juli auf 39° im September ab. Das kann, auf Grund zu geringer Sonneneinstrahlung zum Zeitpunkt der Reife, zu einer unzureichenden Ausprägung der roten Deckfarbe der Äpfel führen (Blanke 2015). Das Ziel von Reflexionsfolien ist es, das bisher ungenutzte Licht aus den Fahrgassen zu nutzen, um durch diffuse (z. B. Lumilys/Extenday) oder gerichtete (z. B. Mylar/Color-up) Reflexion des eingestrahlten Sonnenlichtes eine Verbesserung der Farbentwicklung zu erhalten. Durch die grünen Blätter der Bäume kann es zu Schattierungen auf den Äpfeln kommen, wodurch dann ebenfalls die Sonneneinstrahlung reduziert wird und somit die Farbausprägung negativ beeinflusst werden kann. Nach Lichtmessungen und Modellierung der Lichtverteilung im Apfelbaum mit/ohne Reflexionsfolie (Lumilys) von Weber et al. (2019) soll das bereits von der Mulchfolie in die Baumkrone reflektierte Licht nochmals an den helleren Blattunterseiten reflektiert und damit die ganze Baumkrone aufgehellt werden.

### Spektrale Messungen der Lichtreflexion

Die spektralen Messungen ergaben keine Unterschiede im Kurvenverlauf zwischen den beiden Reflexionsfolien und durch den braunen Boden, der keine Farbe bevorzugt. Bei Gras war dagegen das Reflexionsmaximum bei ca. 750 nm (Abb. 7a,b) in Einklang mit Untersuchungen von Tartachnyk et al. (2006). Die spektrale Lichtmessung des Ap-Horizonts des Bodens (Abb. 7a,b) ergab, dass die Reflexion vom sichtbaren Bereich (20 %) zum infraroten Bereich (28 %) um 8 % leicht zunahm (von 500–700 nm). Das ist vergleichbar mit dem Ergebnis von Lilienthal (2014), bei der die Lichtreflexion bei 500 nm bei 18 % lag und bei 700 nm eine Lichtreflexion von 28 % gemessen wurde und somit um 10 % anstieg. Die Lichtreflexion des Bodens kann variieren und ist abhängig von Bodentyp, Bodenart, organischer Substanz, Bodentextur und Bodenfeuchte.

## Oberflächenrauhigkeit

Sowohl die gerichtete (0°) als auch diffuse (45°) Lichtreflexion (PAR) der weißen Gewebefolie Lumilys nahm mit dem Verschmutzungsgrad ab (Abb. 6a,b). Ursache dafür war die raue Oberfläche der Lumilys (Abb. 10), wodurch die Lichtstrahlen beim Eintreffen auf die Oberfläche diffus reflektiert wurden. Bei der Lumilys im 45°-Winkel reflektierte die saubere Folie 46 % mehr Licht als mit der starken Verschmutzung und bei einem Reflexionswinkel von 0° wurde bei der sauberen Lumilys sogar 68 % mehr Licht als bei der Lumilys mit der starken Verschmutzung reflektiert. Bei der diffusen (45°) Reflexion der Lumilys kam es zu einem leichten Rückgang der Reflexionswerte von 100 % bei 500 nm um 6 % bei 750 nm.

Die unerwartete Zunahme der diffusen Lichtreflexion (45°) der Alufolie mit zunehmender Verschmutzung (Abb. 6c und 8b) konnte durch die Falschfarbenbilder der Rauhigkeitsmessung mit dem Profilometer VR 5200 der Firma Keyence erklärt werden (Abb. 10). Das gleiche gilt für die höhere diffuse (45°) und gerichtete (0°) Lichtreflexion (PAR) der weißen Gewebefolie Lumilys (Abb. 8a) und die gerichtete UV‑B Reflexion (0°) der weißen Gewebefolie mit zunehmendem Verschmutzungsgrad (Abb. 9a).

## Fazit

**Abb. 11:**
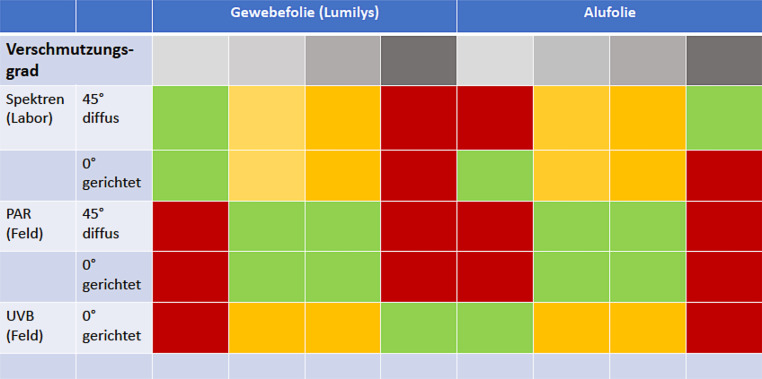
Visualisierung aller Ergebnisse der Lichtreflexionsmessungen im Labor und im Feld (PAR und UV-B): *grün* höchste, *gelbe* mittlere und *rot* geringe Lichtreflexion. Die Graustufen symbolisieren den Verschmutzungsgrad

Die Untersuchungen (Abb. 11) haben gezeigt, dass

1. die weiße Gewebefolie Lumilys eine hohe diffuse Lichtreflexion (45°-Winkel) und die Alufolie eine hohe gerichtete (0°) Reflexion aufwiesen;
2. Verschmutzung nicht generell zu einer Verminderung der Reflexion führte, sondern die diffuse (45°) Lichtreflexion steigern konnte;
3. die gerichtete (0°) UV-B-Reflexion bei der weißen Gewebefolie zunahm, aber bei der Alufolie sank;
4. die Reflexionsfolien bei Verschmutzung noch wirksam sind und sich ihre Lebensdauer verlängert.
